# Structural Characterization of a Polysaccharide from *Gastrodia elata* and Its Bioactivity on Gut Microbiota

**DOI:** 10.3390/molecules26154443

**Published:** 2021-07-23

**Authors:** Jiangyan Huo, Min Lei, Feifei Li, Jinjun Hou, Zijia Zhang, Huali Long, Xianchun Zhong, Yameng Liu, Cen Xie, Wanying Wu

**Affiliations:** 1 Shanghai Research Center for Modernization of Traditional Chinese Medicine, National Engineering Laboratory for TCM Standardization Technology, Shanghai Institute of Materia Medica, Chinese Academy of Sciences, Shanghai 201203, China; huojiangyan@simm.ac.cn (J.H.); mlei@simm.ac.cn (M.L.); s19-lifeifei@simm.ac.cn (F.L.); jinjun_hou@simm.ac.cn (J.H.); zijiazhang@simm.ac.cn (Z.Z.); longhuali@simm.ac.cn (H.L.); zhongxianchun@shu.edu.cn (X.Z.); yameng_liu@simm.ac.cn (Y.L.); 2 University of Chinese Academy of Sciences, Beijing 100049, China

**Keywords:** *Gastrodia elata*, structural characterization, polysaccharide, gut microbiota

## Abstract

A novel homogeneous polysaccharide named **GEP-1** was isolated and purified from *Gastrodia elata* (*G. elata*) by hot-water extraction, ethanol precipitation, and membrane separator. **GEP-1**, which has a molecular weight of 20.1 kDa, contains a polysaccharide framework comprised of only glucose. Methylation and NMR analysis showed that **GEP-1** contained 1,3,6-linked-α-Glc*p*, 1,4-linked-α-Glc*p*, 1,4-linked-β-Glc*p* and 1,4,6-linked-α-Glc*p*. Interestingly, **GEP-1** contained citric acid and repeating *p*-hydroxybenzyl alcohol as one branch. Furthermore, a bioactivity test showed that **GEP-1** could significantly promote the growth of *Akkermansia muciniphila* (*A. muciniphila*) and *Lacticaseibacillus paracasei* (*L.* *paracasei*) strains. These results implied that **GEP-1** might be useful for human by modulating gut microbiota.

## 1. Introduction

*Gastrodia elata* (*G. elata*), from the family Ochidaceae, is a traditional Chinese medicine, which is widely used in the clinic. Modern pharmacological research shows that *G. elata* has a variety of biological activities, such as anti-convulsion [1,2], improving memory [3], preventing senescence [4], neuroprotection [5], etc. [6,7,8,9]. In the past, more attention was paid to the small molecular compounds in *G. elata*, including gastrodin, parishin, phenolic compounds, 4-hydroxybenzyl alcohol, β-sitosterol and so on [10,11,12,13,14]. In recent years, with the progress of separation, purification and detection technology, polysaccharide macromolecules in *G. elata* are receiving increasing attention. *G. elata* contains more than 10% polysaccharide components, which also show a variety of biological activities [15,16,17,18,19]. For example, Xie et al. reported that polysaccharides of *G. elata* can improve the learning and memory ability of D-galactose–induced aging mice to improve the activity of enzymes related to oxidative metabolism in the body [20]. In addition, polysaccharides of *G. elata* are shown to increase the number of brain-derived neurotrophic factor (BDNF)-positive cells and stem cell factor (SCF)-positive cells and decrease the average gray value, suggesting that polysaccharides from *G. elata* (PGB) have neuroprotective effects by up-regulating BDNF and SCF expression in brain tissues around ischemic lesions [21]. Therefore, it is of great significance to study the isolation, purification, and biological activities of polysaccharides from *G. elata*.

Since oral polysaccharides are hardly absorbed, they may not directly act on the target organs in vivo to produce biological effects. Although polysaccharides cannot be directly absorbed by the human body, they can be decomposed and utilized by gut microbiota to produce a series of bioactive metabolites [22]. In recent years, with the deepening of research, more and more researchers believe that polysaccharides can affect the structure and function of gut microbiota and play a regulatory role in human health by remodeling intestinal microecology or intestinal homeostasis [23,24,25]. For example, *Lycium barbarum* polysaccharides can promote the production of short-chain fatty acids and modulate the composition of the gut microbiota, increasing the relative abundances of Bacteroidaceae, Lactobacillaceae, Prevotellaceae and Verrucomicrobiaceae, which are positively associated with immunomodulatory traits [26]. In 2013, Zhang and his co-workers reported that polysaccharide from the fungus *Flammuliana* *velutipes* improves colitis via regulation of colonic microbial dysbiosis and inflammatory responses [27]. In conclusion, gut microbiota have become an important bridge for polysaccharides to exert their biological activities in vivo.

Based on the above literature reports, we carried out related research on the ability of polysaccharide of *G. elata* to promote the growth of probiotics. First, the polysaccharides from *G. elata* were extracted and separated, and a homogeneous polysaccharide named GEP-1 was obtained. The structure of GEP-**1** was characterized by HPGPC, PMP-HPLC (1-phenyl-3-methyl-5-pyrazolone pre-column derivatization prior to HPLC), LC/MS, FT-IR, NMR, and SEM methods. Then, two gut microbiota, *Akkermansia muciniphila* (*A. muciniphila*) and *Lacticaseibacillus paracasei* (*L.* *paracasei*), were selected to evaluate the activity of GEP-1. GEP-1 demonstrated promoting effects on the growth of the two selected gut microbiota. Therefore, in this paper, we will report the isolation, purification and structural characterization of GEP-1, as well as its growth promoting effect on gut microbiota.

## 2. Results and Discussion

### 2.1. Extraction, Isolation and Purification of GEP-1

**Figure 1:**
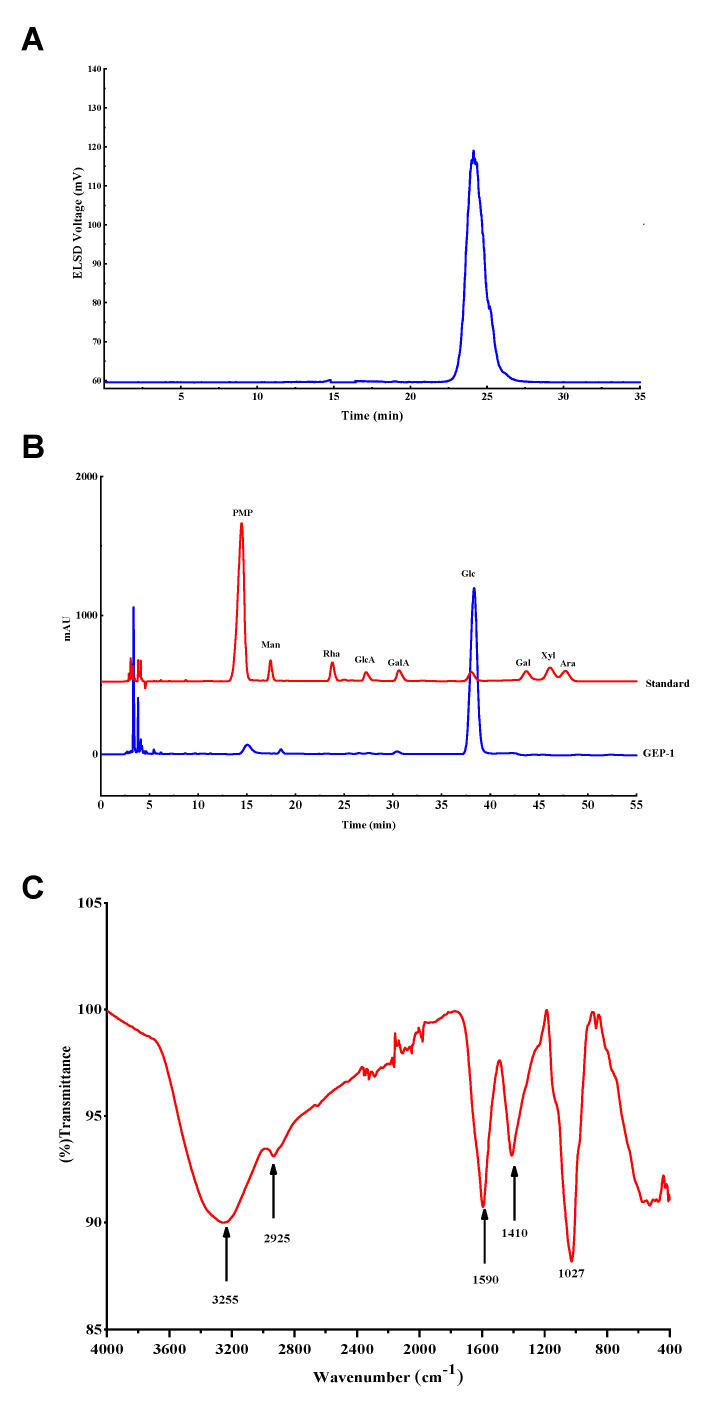
(**A**): HPGPC of GEP-1; (**B**): HPLC chromatograms of PMP derivatives; (**C**): FT-IR spectrum of GEP-1.

Crude polysaccharides GEP50 (200 g, yield: 5.56%) were extracted from the rhizome of *G. elata* (3.6 kg) by water extraction and alcohol precipitation. Then GEP50 (180 g) was dissolved in water (2.5 L), separated by the membrane separation equipment with 10 kDa membrane and eluted with H_2_O to obtain a homogeneous polysaccharide, GEP-1 (20 g, yield: 11.11%). The elution curve of is displayed in Figure S1. As shown in Figure 1A, GEP-1 showed a symmetrical peak, indicating it was a homogeneous polysaccharide and the molecular weight was 20.15 kDa according to the standard curve. Furthermore, the protein content of GEP-1 was 0.78 ± 0.03% tested using a BCA reagent kit.

### 2.2. Monosaccharide Composition, Linkage Pattern and CA and Repeated HA Analysis

The monosaccharide composition of GEP-1 was detected by PMP pre-column derivative method. The HPLC result (Figure 1B) showed that GEP-1 was composed of Glc compared with standards. The linkage patterns of glycosidic residues in GEP-1 were determined by methylation method and GC-MS analysis.

The result (Table 1) demonstrated that fully *O*-methylated GEP-1 was comprised of 2,3,4,6-Me_4_-Glc*p*, 2,3,6-Me_3_-Glc*p*, 2,3,4-Me_3_-Glc*p*, 2,3-Me_2_-Glc*p* and 2,6-Me_2_-Glc*p* with the molar ratio of 2.23:9.69:4.93:1.08:2.07. Interestingly, the signals around 7.0 ppm and 2.0 ppm in ^1^H-NMR and the signals between 110 ppm and 180 ppm in the ^13^C-NMR spectra of GEP-1 indicated that non-polysaccharides constituents might be linked to polysaccharide part in GEP-**1**. Also, non-polysaccharide constituents might be *p*-HA and CA based on the NMR signals and reported structures of constituents in *G. elata* [28,29]. In order to understand the *p*-HACA in GEP-1, GEP-1 (3 mg) was hydrolyzed with 2 M TFA to obtain the full hydrolysates. The LC-MS results (Figure S2 and Table 2) showed that *m/z* 193.0343 [CA + H]^+^, *m/z* 107.0489 [*p*-HA − H_2_O + H]^+^, and *m/z* 163.0597 [Glc − H_2_O + H]^+^ confirmed the existence of CA and *p*-HA. Interestingly, consecutive neutral losses of 106.04 Da between *m/z* 107.0489 [HA − H_2_O + H]^+^, *m/z* 213.0913 [2HA − H_2_O + H]^+^, *m/z* 319.1326 [3HA − H_2_O + H]^+^, *m/z* 425.1745 [4HA − H_2_O + H]^+^, *m/z* 531.2161 [5HA − H_2_O + H]^+^, *m/z* 637.2576 [6HA − H_2_O + H]^+^, and *m/z* 743.3001 [7HA − H_2_O + H]^+^ in positive mode, and also among *m/z* 105.0349 [HA − H_2_O − H]^−^, *m/z* 211.0766 [2HA − H_2_O − H]^−^, *m/z* 317.1182 [3HA − H_2_O − H]^−^, *m/z* 423.1605 [4HA − H_2_O − H]^−^, *m/z* 529.2029 [5HA − H_2_O − H]^−^, *m/z* 635.2458 [6HA − H_2_O − H]^−^, *m/z* 741.2882 [7HA − H_2_O − H]^−^, *m/z* 847.3228 [8HA − H_2_O −H]^−^, *m/z* 953.3691 [9HA − H_2_O − H]^−^ and *m/z* 1059.4114 [10HA − H_2_O − H]^−^ in negative mode suggested the repeating *p*-HA unit in GEP-1. Furthermore, GEP-1 was hydrolyzed using 4 mol/L of NaOH in 50% methanol and acidized by HCl to determine the content of CA. Finally, the content of CA in GEP-1 was about 3.93% by comparing the area of its full alkaline hydrolysates with the CA standard.

### 2.3. FT-IR Analysis

The FT-IR spectrum of GEP-1 illustrated in Figure 1C showed strong absorption bands at 3300 and 2900 cm^−1^. These bands were attributed to the stretching vibrations of OH and CH groups, respectively. Meanwhile, the intense peaks at 1600 and 1400 cm^−1^ correspond to the asymmetric and symmetric C=O or C=C stretching vibrations. The absorption bands observed at 1030 and 600 cm^−1^ suggested that GEP-1 comprised a pyranose structure. In general, all the spectroscopic bands identified were characteristics of polysaccharides, *p*-HA and CA.

### 2.4. NMR Analysis

**Figure 2:**
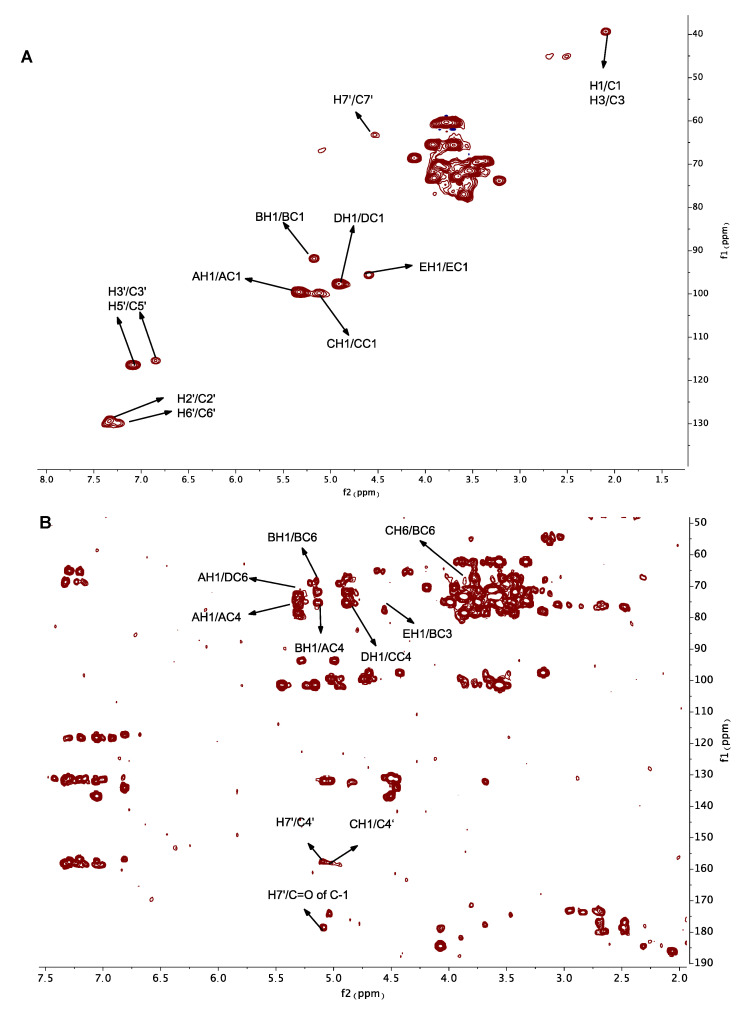
HSQC (**A**) and HMBC (**B**) spectra of GEP-1.

NMR plays an essential role in the structural characterization of polysaccharides. In this study, ^1^H, ^13^C, HSQC and HMBC spectra (Figure 2) of GEP-1 were recorded. Due to the existence of repeating *p*-HA and CA in GEP-1, the first step in analyzing the NMR spectra was the identification of repeating *p*-HA and CA. Based on the literature [30], peaks at 2.07/39.50 ppm, 7.29/129.38 ppm or 7.23/130.15 ppm, 6.85/115.56 ppm or 7.09/116.56 ppm, and 5.14/67.94 ppm in HSQC were attributed to H1/C1 (H3/C3), H2′/C2′ (H6′/C6′), H3′/C3′(H5′/C5′), and H7′/C7′, and signals at 68.35 ppm, 129.55 ppm, 157.55 ppm, 179.56 ppm, 182.52 ppm and 181.65 ppm in the ^13^C-NMR spectra were attributed to C2, C1′, C4′, C=O of C-1, C=O of C-2 and C=O of C-3 of *p*-HACA and repeated *p*-HA in GEP-1. And the signals at 5.14/179.56 ppm (H7′/C=O of C-1) and 5.14/157.55 ppm (H7′/C4′) in HMBC spectrum indicated C=O of C-1 of CA was linked to H-7′ of HA, H-7′ of HA linked to C-4′ of HA, confirming the existences of *p*-HA CA and repeated *p*-HA.

Secondly, the identification of anomeric carbon and anomeric proton signals were analyzed. Due to the fact 1,4-Glc*p* was the most abundant glycosidic residue according to the methylation result the most intense anomeric proton signal at 5.33 ppm in the ^1^H-NMR spectrum was assigned to H1 of 1,4-Glc*p*, and the signal at δ_H_ 5.33 ppm was corresponding to δ_C_ 99.67 ppm in the HSQC spectrum, so the peak at 5.33/99.67 ppm was assigned to H1/C1 of 1,4-Glc*p*. Therefore, based on the results of methylation analysis, the NMR spectra and the literature, the anomeric signals at 5.33/99.67 ppm, 5.18/91.93 ppm, 5.12/100.00 ppm, 4.91/97.90 ppm and 4.60/95.73 ppm were distributed to⟶4)-α-Glc*p*-(1⟶, ⟶3,6)-α-Glc*p*-(1⟶, ⟶4,6)-α-Glc*p*-(1⟶, ⟶6)-β-Glc*p*-(1⟶ and β-Glc*p*-(1⟶, and named as residue A, B, C, D and E, respectively. Based on the literature and 2D NMR spectra, the signals at 3.56/73.15 ppm, 3.89/74.93 ppm, 3.89/73.37 ppm, 3.89/74.93 ppm and 3.70/61.87 ppm were assigned to H2/C2, H3/C3, H4/C4, H5/C5 and H6/C6 of 1,4-Glc*p*.

**Figure 3:**
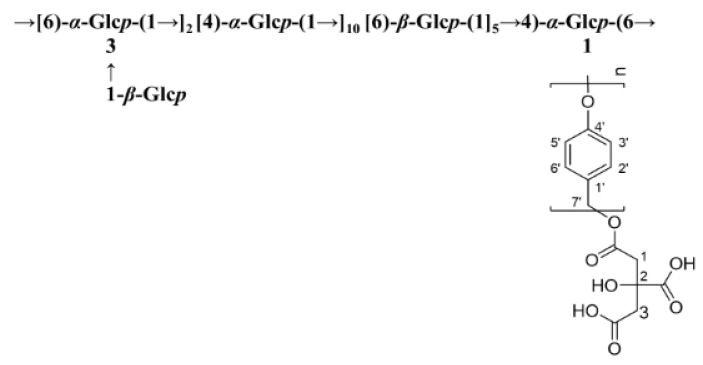
The putative repeating unit of **GEP-1**.

The detailed chemical shifts of GEP-1 are shown in Table 3. The linkage sequences of residues in GEP-1 were speculated by signals in HMBC spectrum. The peaks at 3.90/67.21 ppm (CH6/BC6), 4.60/76.34 ppm (EH1/BC3), 5.33/68.76 ppm (AH1/DC6), 5.18/73.37 ppm (BH1/AC4), 4.91/74.33 ppm (DH1/CC4) indicated that O-6 of residue C was linked to C-6 of residue B, O-1 of residue E linked to C-3 of residue B, O-1 of residue A linked to linked to C-6 of residue D, O-1 of residue B linked to C-4 of residue A, O-1 of residue D linked to C-4 of residue C. Also, the peaks at 5.33/73.37 ppm (AH1/AC4), 5.18/67.21 ppm (BH1/BC6) and 4.91/68.76 ppm (DH1/DC6) illustrated the linkage between O-1 of residue A and C-4 of residue A, the linkage between O-1 of residue B and C-6 of residue B and the linkage between O-1 of residue D and C-6 of residue D, indicating that residue A, residue B and residue D were repeated in the structure of GEP-1. Further, the signal at 5.10/157.55 ppm (CH1/C4’) revealed that O-1 of residue C was linked to C4’ of repeated *p*-HA. Together, the possible repeating unit of GEP-1 is shown in Figure 3.

### 2.5. SEM Analysis

**Figure 4:**
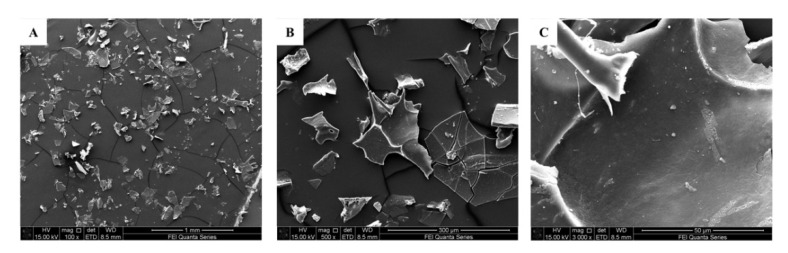
SEM images of **GEP-1** at different magnifications (**A**: 100 ×; **B**: 500 ×; **C**: 3000 ×).

The surface morphologies of GEP-1 were observed through SEM at 100 ×, 500 × and 3000 × magnifications. The SEM images of GEP-1 were displayed in Figure 4, GEP-1 exhibited an irregular, laminated, wrinkled, sheet-like morphology with a little rough surface, which was accordance with its branched structure.

### 2.6. GEP-1 Induced the Growth A. muciniphila and L. paracasei

**Figure 5:**
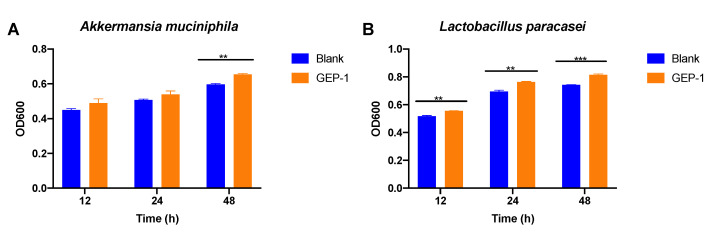
(**A**): The growth of *A. muciniphila* in the medium containing 1 mg/mL GEP-1 for 12, 24 and 48 h; (**B**): The growth of *L. paracasei* in the medium containing 1 mg/mL GEP-1 for 12, 24h and 48 h. (*n* = 3, by student t test, ** *p* < 0.01, *** *p* < 0.001.).

To test whether GEP-1 could have effect on *A. muciniphila* and *L. paracasei* strains, the two strains were cultured supplemented with 1 mg/mL of GEP-1. As shown in Figure 5A, it was showed that the growth of *A. muciniphila* was promoted by polysaccharide GEP-1, and significant promotion was detected at 48 h. In Figure 5B, GEP-1 significantly induced the growth of *L. paracasei* at 12 h, 24 h and 48 h. These results indicated that GEP-1 had bioactivity on human gut probiotic *A. muciniphila* and *L. paracasei in vitro*.

Previous studies suggested that most homogeneous polysaccharides from *G. elata* consisted only of glucose and have a main chain of 1-4 linked glycosidic bonds. GEP-1 also had a backbone of 1,4-linked glucose and 1,6-linked glucose. But what was special was that GEP-**1** contained a branch of CA and repeating *p*-HA. Most of the isolated glucans exhibited anti-cancer, antioxidant and immunological activities [31]. In this experiment, GEP-1 obviously induced the growth of *A. muciniphila* and *L. Paracasei*, and the regulatory effects of polysaccharides from *G. elata* on two bacteria from human intestinal flora were demonstrated.

## 3. Materials and Method

### 3.1. Materials

The dried rhizomes of *G. elata* were obtained from Zhaotong (Yunnan Province, China) and authenticated by Menghua Tian from Zhaotong Tama Research Institute of Yunnan Province (China). Monosaccharide standards, glucose (Glc), mannose (Man), rhamnose (Rha), glucuronic acid (GlcA), galacuronic acid (GalA), galactose (Gal), xylose (Xyl) and arabinose (Ara), and citric acid (CA) were purchased from Sigma (St. Louis, MO, USA). DEAE-52 were purchased from Whatman (Kent, UK). BCA reagent kit and MRS medium was purchased from Dalian Meilun Biotech Co., Ltd. (Dalian, China). Defined medium was in-house prepared. *A. muciniphila* strain ATCC BAA-835 and *L. paracasei* were obtained from BeiNa Chuanglian Biotechnology Co., Ltd. (Beijing, China).

### 3.2. Extraction, Isolation and Purification of GEP-1

The dried rhizomes of *G. elata* (3.6 kg) were first crushed to fine particles and extracted with 30 volumes of distilled water at 70 °C for 4 h for three times. The concentrated water extract was precipitated with 95% ethanol to a final concentration of 50% and deproteinized with Sevage method [32] to obtain crude polysaccharide (**GEP50**). **GEP50** was applied on the membrane separation equipment (Shanghai Mosu Science Equipment Co., Ltd., Shanghai, China) equipped with a 10 kDa membrane and eluted with H_2_O. Finally, a homogeneous polysaccharide GEP-1 was obtained.

### 3.3. Estimation of Homogeneity and Molecular Weight

The homogeneity and molecular weight of GEP-1 was estimated by HPGPC using an Agilent Technologies system (Santa Clara, CA, USA) equipped with an ELSD. The sample (2 mg/mL and 20 µL) was applied to series TSK G5000 and G3000 columns and eluted with H_2_O at 0.8 mL/min. Commercially available dextrans (Mw: 2.70, 5.25, 9.75, 13.05, 64.65, 135.30, 300.60 kDa) were used as standard molecular markers for the construction of the calibration curve. The molecular weight was estimated based on the calibration curve.

### 3.4. Analysis of Composition

The carbohydrate content was determined by phenol-sulfuric acid colorimetric method using glucose as standard. The protein content was measured using BCA reagent kit. Based on the previous studies that investigated small molecules, many of which contained *p*-hydroxylbenzyl alcohol (*p*-HA) and/or CA [10], CA and repeated *p*-HA might be present in the polysaccharides from *G. elata*. Therefore, the sample was fully hydrolyzed with 2 M TFA at 110 °C for 5 h and then dissolved in 50% methanol and components in the hydrolysate were identified by LTQ-Orbitrap Velos Pro hybrid mass spectrometer (Thermo Fisher, Scientific, Carlsbad, CA, USA). The chromatographic separation was carried out on an HSS T3 column (2.1 × 150 mm i.d., particle size 1.8 µm, Waters, Milford, CT, USA) operated at 30 °C. The mobile phase consisted of 0.1% formic acid (FA) in water (A) and acetonitrile (B) with a flow rate of 0.3 mL/min. The gradient elution was as follows: 0–12 min, 0–100% B; 12-15 min, 100% B; 15–20 min, 100%–0% B. Later, GEP-1 was hydrolysed using 4 M NaOH in 50% methanol (2 mL) for 3 h at room temperature, neutralized by HCl, and then the content of citric acid in GEP-1 was determined by AQUITY UPLC system (Waters) equipped with a C18 column using CA as the standard solution. The binary mobile phase consisted of 0.1% FA in 90% water (A) and 0.1% FA in 90% acetonitrile (B). The elution gradient was set as follows: 0–6 min, 0 %–100%B.

### 3.5. Monosaccharide Composition

The monosaccharide composition of the polysaccharide was determined by PMP-derivative method [33]. 3 mg of GEP-1 was hydrolyzed in 2 mL of 2 mol/L trifluoracetic acid (TFA) solution at 110 °C for 5 h. After TFA fully being eliminated by nitrogen, the hydrolysate was dissolved in 500 µL ammonium solution (25%) and mixed with 500 µL of 0.5 mol/L PMP-methanol solution, and the reaction was conducted at 70 °C for 2 h. After the elimination of ammonia by nitrogen, the reaction product was dissolved in 500 µL water and extracted with chloroform. Finally, the supernatant was tested on Agilent 1260 HPLC equipped with an Agilent C18 column (4.6 × 200 mm, 5 µm). The sample was eluted with 20 mM ammonium acetate in water (82.5%) and acetonitrile (17.5%) at 30 °C with 1 mL/min flow rate.

### 3.6. Fourier Transform Infrared (FT-IR) Spectroscopy

2 mg sample dissolved in chloroform was added on the KBr window and dried under infrared light, and then measured by Thermo Nicolet iS5 FTIR spectrometer in the middle infrared range of 4000—400 cm^−1^.

### 3.7. Methylation Analysis

The methylation analysis of GEP-1 was referred to the Hakomori method based on the previous literature [34]. The sample (20 mg) was per-*O*-methylated by repeated reaction with NaH-DMSO and methyl iodide until the methylation was completed. Then, the residue was hydrolyzed by 2 M TFA at 110 °C for 5 h. After that, the sample was reduced with NaBD4 (25 mg) and acetylated with acetic anhydride/pyridine. Finally, the partially methylated alditol acetates (PMAAs) were analyzed by GC-MS (Agilent 7890A).

### 3.8. NMR Analysis

GEP-1 (60 mg) was dissolved in deuterium oxide (D_2_O, 500 µL), and exchanged hydrogen with deuterium for three times by repeated freeze-drying. After lyophilization, the sample was dissolved in D_2_O (500 µL), centrifuged and transferred into an NMR tube. The NMR spectra (^1^H, ^13^C, HSQC and HMBC) were obtained by a 600 MHz Bruker spectrometer (Bruker, Ettlingen, Germany).

### 3.9. Scanning Electron Microscopy (SEM) Analysis

The microscopic features of GEP-1 were observed by a scanning electron microscope (SEM) according to a previously reported method [35]. In brief, GEP-1 was ground into powder and distributed evenly on a silicon wafer using double-sided adhesive tape (1 cm × 1 cm). Subsequently, the wafer was sprayed with gold powder using a sputter coater. Finally, SEM images of GEP-1 at different magnifications were recorded by SEM (Quanta 250, FEI, Hillsboro, OR, USA).

### 3.10. Strains

*A. muciniphila* and *L. paracasei* were cultured in MRS medium or defined medium. Bacteria were seeded (OD600 = 0.1) in the medium containing 1 mg/mL of GEP-1. The culture medium was then collected for the determination of OD600 through microplate reader.

## 4. Conclusions

A homogeneous polysaccharide with molecular weight 20.15 kDa and named GEP-1 was isolated and purified from *G. elata*. The monosaccharide composition of **GEP-1** contained only glucose. The backbone of GEP-**1** was composed of →[6)-α-Glc*p*-(1→]_2_ [4)-*α*-Glc*p*-(1→]_10_ [6)-β-Glc*p*-(1]_5_→4)-α-Glc*p*-(6→, with three branches of β-Glc*p* and CA-repeating *p*-HA attached to the backbone chain at O-3 position of 1,3,6-linked α-Glc*p* and O-1 position of 1,4,6-linked α-Glc*p*. Bioactivity tests showed that GEP-1 could promote the growth of *A. muciniphila* and *L. paracasei* strains. These results imply that GEP-1 could be employed to modulate human gut microbiota for the treatment of diseases.

## Figures and Tables

**Table 1:** Linkage patterns analysis of GEP-1.

Methylated Sugars	Linkages	Major Mass Fragments (*m/z*)	Molar Ratio
2,3,4,6-Me_4_-Glc	1-linked-Glc	43, 71, 87, 102, 118, 129, 145, 161, 162, 205	2.23
2,3,6-Me_3_-Glc	1,4-linked-Glc	43, 59, 71, 87, 102, 118, 129, 162, 233	9.69
2,3,4-Me_3_-Glc	1,6-linked-Glc	43, 59, 87, 99, 101, 118, 129, 162, 189, 233	4.93
2,3-Me_2_-Glc	1,4,6-linked-Glc	43, 59, 85, 102, 118, 127, 162, 201, 161	1.08
2,6-Me_2_-Glc	1,3,4-linked-Glc	43, 59, 87, 118, 129, 160, 185, 305	2.07

**Table 2:** LC-MS results of non-polysaccharide constituents in GEP-1 hydrolysate.

Name	Molecular Formula	Theoric Mass	t_R_ (min)	*m*/*z* Experimental	pos/neg	Error (ppm)
CA	C_6_H_8_O_7_	192.0265	2.72	193.0343	[M + H]^+^	−0.047
1HA	C_7_H_8_O_2_	124.0519	1.12	107.0489	[M − H_2_O + H]^+^	−2.535
2HA	C_14_H_14_O_3_	230.0937	8.44	213.0913	[M − H_2_O + H]^+^	1.473
3HA	C_21_H_20_O_4_	336.1356	8.86	319.1326	[M − H_2_O + H]^+^	-0.894
4HA	C_28_H_26_O_5_	442.1775	9.16	425.1745	[M − H_2_O + H]^+^	−0.554
5HA	C_35_H_32_O_6_	548.2193	9.40	531.2161	[M − H_2_O + H]^+^	−1.018
6HA	C_42_H_38_O_7_	654.2612	9.52	637.2576	[M − H_2_O + H]^+^	−1.311
7HA	C_49_H_44_O_8_	760.3031	9.58	743.3001	[M − H_2_O + H]^+^	−0.377
Glc	C_6_H_12_O_6_	180.0628	1.02	163.0597	[M − H_2_O + H]^+^	−2.207
CA	C_6_H_8_O_7_	192.0265	2.75	191.0196	[M − H]^−^	5.293
Glc	C_6_H_12_O_6_	180.0628	1.02	179.0561	[M − H]^−^	0.160
1HA	C_7_H_8_O_2_	124.0519	1.12	105.0349	[M − H_2_O + H]^−^	2.493
2HA	C_14_H_14_O_3_	230.0937	8.44	211.0766	[M − H_2_O − H]^−^	0.839
3HA	C_21_H_20_O_4_	336.1356	8.92	317.1182	[M − H_2_O − H]^−^	−0.403
4HA	C_28_H_26_O_5_	442.1775	9.16	423.1605	[M − H_2_O − H]^−^	0.656
4HA	C_28_H_26_O_5_	442.1775	9.16	411.1606	[M − CH_2_O − H]^−^	1.113
5HA	C_35_H_32_O_6_	548.2193	9.13	529.2029	[M − H_2_O − H]^−^	1.681
6HA	C_42_H_38_O_7_	654.2612	9.38	635.2458	[M − H_2_O − H]^−^	2.956
7HA	C_49_H_44_O_8_	760.3031	9.49	741.2882	[M − H_2_O − H]^−^^-^	3.201
8HA	C_56_H_50_O_9_	866.3449	9.73	847.3228	[M − H_2_O − H]^−^	0.140
9HA	C_63_H_56_O_10_	972.2868	9.76	953.3691	[M − H_2_O − H]^−^	−0.447
10HA	C_70_H_62_O_11_	1078.4287	9.85	1047.4118	[M − CH_2_O − H]^−^	0.476
10HA	C_70_H_62_O_11_	1078.4287	9.85	1059.4114	[M − H_2_O − H]^−^	0.008

**Table 3:** Chemical shifts of main residues of **GEP-1**.

Code	Residues	Chemical Shifts (ppm)					
		H1/C1	H2/C2	H3/C3	H4/C4	H5/C5	H6/C6
A	⟶4)-α-Glc*p*-(1⟶	5.33/99.67	3.56/73.15	3.89/74.93	3.89/73.37	3.89/74.93	3.70/61.87
B	⟶3,6)-α-Glc*p*-(1⟶	5.18/91.93	3.64/73.0	3.76/76.34	3.39/71.67	3.81/71.91	3.63/67.21
C	⟶4,6)-α-Glc*p*-(1⟶	5.10/100.00	3.58/73.42	3.90/74.93	3.67/74.33	3.83/71.78	3.90/66.07
D	⟶6)-β-Glc*p*-(1⟶	4.91/97.90	3.38/76.4	3.63/77.4	3.45/72.6	3.63/77.7	3.74/68.76
E	β-Glc*p*-(1⟶	4.60/95.73	3.19/73.96	3.72/72.89	3.34/69.37	3.55/74.57	3.85/60.52

## Data Availability

Data will be provided upon request.

## Supplementary Materials

The following are available online, Figure S1: The BPI chromatogram (A) and MS/MS spectra of 10HA in **GEP-1** hydrolysate in negative mode (B), Figure S2: The mass spectra of PMAAs derived from **GEP-1**, Figure S3: ^1^H- and ^13^C-NMR spectra of **GEP-1**.

## Sample Availability

Samples of the **GEP-1** is available from the authors.
