# Supplementary material for: Structural Characterization of a Polysaccharide from Gastrodia elata and Its Bioactivity on Gut Microbiota
Source: Molecules. 2021 Jul 23;26(15):4443. doi: 10.3390/molecules26154443 (PMC8348156; doi:10.3390/molecules26154443)

Pages of Supplementary Material for Article

Structural characterization of a polysaccharide from *Gastrodia elata* and its bioactivity on gut microbiota

by

Jiangyan Huo [1,2,†], Min Lei [1,2,†], Feifei Li [1,2], Jinjun Hou [1,2], Zijia Zhang [1,2], Huali Long [1,2], Xianchun Zhong [1,2], Yameng Liu [1,2], Cen Xie [1,2,*] and Wanying Wu [1,2,*]

[1] Shanghai Research Center for Modernization of Traditional Chinese Medicine, National Engineering Laboratory for TCM Standardization Technology, Shanghai Institute of Materia Medica, Chinese Academy of Sciences, Shanghai 201203, China

[2] University of Chinese Academy of Sciences, Beijing 100049, China

* Correspondence: xiecen@simm.ac.cn (C.X.); wanyingwu@simm.ac.cn (W.W.)

† These authors contributed equally to this work.

SM 1 **Figure 1**

The BPI chromatogram (A) and MS/MS spectra of 10HA in GEP-1 hydrolysate in negative mode **(B).**

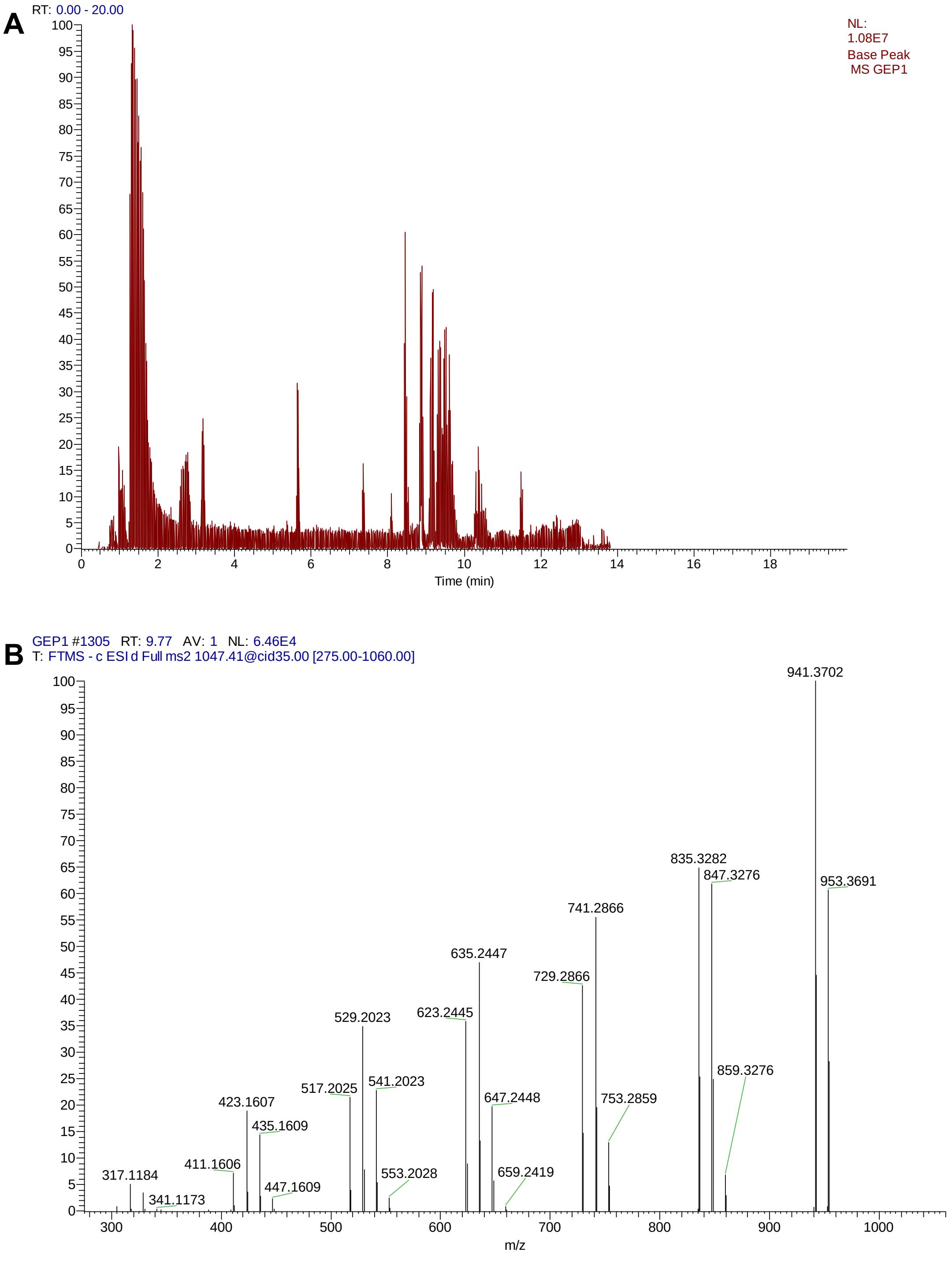

SM 2 **Figure 2**

The mass spectra of PMAAs derived from **GEP-1**.

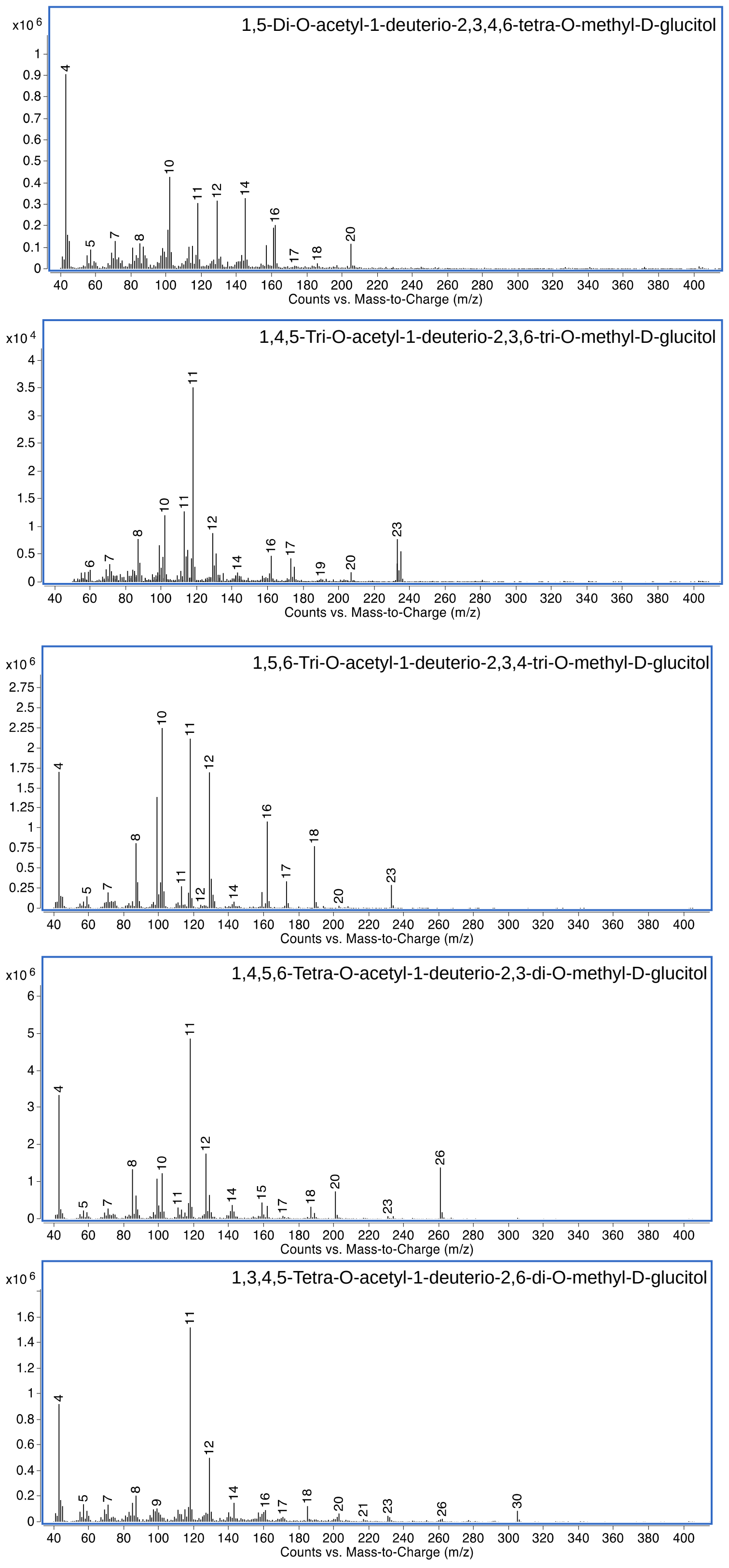

SM 3 **Figure 3**

^{1}H and ^{13}C NMR spectra of **GEP-1**.

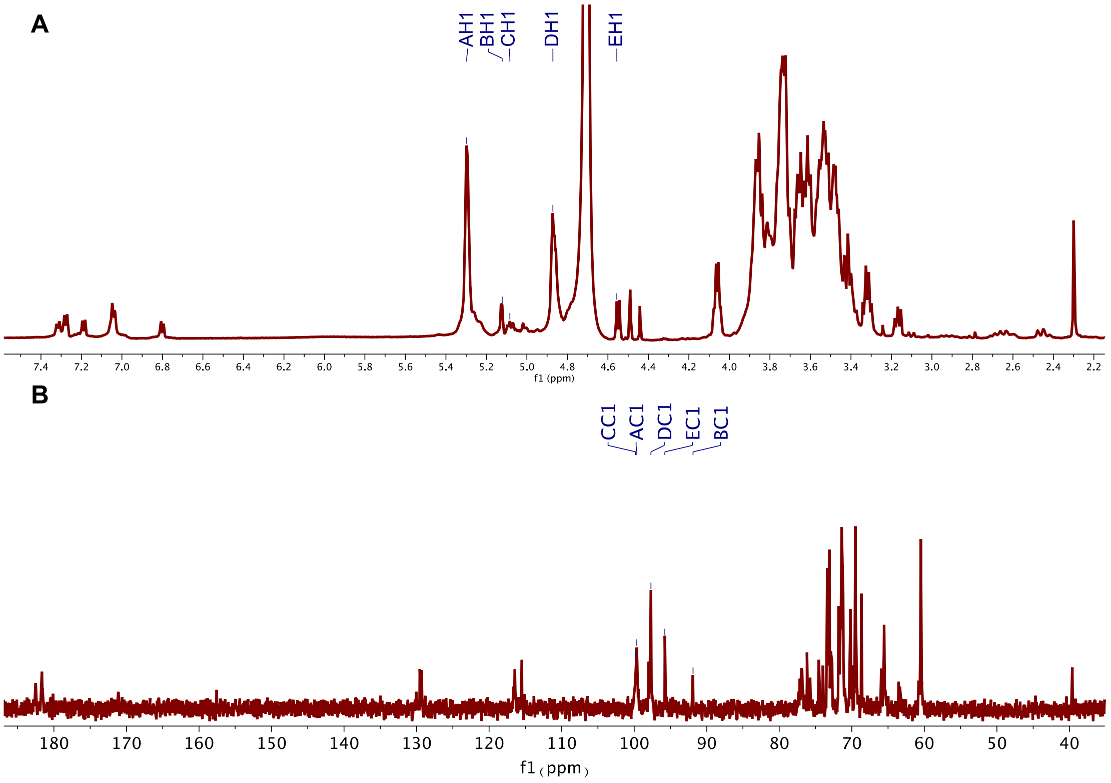

Supplement: Supplementary file 1 [file molecules-26-04443-s001.zip › molecules-1249031-supplementary.pdf]
